# Assessment of the Kernel Gram Matrix Representation of Data in Order to Avoid the Alignment of Chromatographic Signals

**DOI:** 10.3390/molecules26030621

**Published:** 2021-01-25

**Authors:** Ivana Stanimirova, Michal Daszykowski

**Affiliations:** Institute of Chemistry, University of Silesia in Katowice, 9 Szkolna Street, 40-006 Katowice, Poland

**Keywords:** chemical fingerprints, kernel trick, Gram PCA, warping, preprocessing, peak shifts, second-order data, diode-array detector, multichannel detector

## Abstract

This article discusses the possibility of exploratory data analysis of samples described by second-order chromatographic data affected by peak shifts. In particular, the potential of the kernel Gram matrix representation as an alternative to the necessary and time-consuming alignment step is evaluated. It was demonstrated through several simulation studies and comparisons that even small peak shifts can be a substantial source of data variance, and they can easily hamper the interpretation of chromatographic data. When peak shifts are small, their negative effect is far more destructive than the impact of relatively large levels of the Gaussian noise, heteroscedastic noise, and signal’s baseline. The Gram principal component analysis approach has proven to be a well-suited tool for exploratory analysis of chromatographic signals collected using the diode-array detector in which sample-to-sample peak shifts were observed.

## 1. Introduction

Chromatographic techniques are used widely to analyze and characterize complex samples, systems, and different processes. Their considerable popularity is related to their large potential to separate individual mixture components. When separation is effective, chemical components can be further identified and quantified. In a given chromatographic system, several factors drive its resolution power and retention of analytes. The retention of a molecule is a characteristic parameter, which relies on intrinsic molecular properties and the strength of interactions observed among the analyte molecules along with stationary and mobile phase components. In practice, all of these interactions affect the elution time, whereas the increase of resolution in a chromatographic system is associated with some modifications of parameters that influence the strength of these interactions. For instance, in the high-performance liquid chromatography (HPLC), one can modify the chemical composition of the mobile phase either statically or dynamically. These are two opposing possibilities giving rise to either isocratic or gradient separations. The isocratic separation assumes that the mobile phase constituents and their proportions are maintained constant during the chromatographic run, whereas, in the gradient variant, their proportions are dynamically modified over time. The flow rate of mobile phase, pH, and the concentration of the mobile phase modifier are some additional parameters that influence the retention properties of any chromatographic system to a large extent.

Moreover, the column temperature is also a relevant parameter. Other possibilities to increase or tune chromatographic separation arise from selecting very different types of stationary phases. Yet, another option is to handle the resolution issue using the orthogonal chromatographic systems, which offer other retention mechanisms. Finally, the effective separation of complex mixtures may require two-dimensional or N-dimensional separations, i.e., coupling chromatographic systems that are different in terms of retention mechanisms and include selective and/or complementary detectors.

Currently, coupling of various chromatographic systems is straightforward and frequently very beneficial. Nonetheless, essential analytical information is often extracted from data that were recorded using advanced multichannel detectors. A unique advantage of the multichannel detectors, such as a diode-array detector, multiple fluorescence detector, or mass spectrometry is based on recording the existing differences in physicochemical properties of individual mixture components (absorption differences in the UV and/or Vis spectral range, ion velocity, and specific emission spectra), referred to as the second-order advantage in the literature [1,2]. Compared to the classic mono-channel counterparts, the subsequent portions of eluent are characterized by a multichannel detector to a larger extent in addition to the chromatographic separation that is carried out along the time domain. The resulting chromatographic data are comprehensive in nature. They are represented in a form of a second-order data (a matrix) for a single sample and have a three-way structure for a set of samples. Then, chemometric analysis using the multivariate curve resolution (MCR) [3], methods and multiway methods such as parallel factor analysis (PARAFAC) and N-way partial least squares regression (N-PLS) is usually performed in order to extract information about pure mixture components or to solve the second-order calibration problem [4].

Chemical fingerprinting is the most explored concept in the characterization of complex samples and monitoring of different systems and processes. Chemical fingerprinting, combined with the chemometric methods, is highly valued in the non-targeted metabolomic analysis and in quality control of traditional medicines. In general, a chemical fingerprint is an analytical signal, reflecting the chemical content of a sample to the most considerable possible extent. In theory, it uniquely describes a sample as human fingerprints describe a personal identity. Chromatographic techniques, by definition, can provide very diverse chemical information about samples, and, therefore, they are well-suited for fingerprinting purposes. When chromatographic separation is successful, chromatograms contain a large number of peaks. Their locations, heights, and shapes are these unique descriptive features that help to differentiate/identify samples. Chemical fingerprinting concerns the exploration of chemical space in which multichannel detectors are beneficial because they increase the diversity and orthogonality of information. Chromatographic separation, combined with multichannel detection and chemometric modeling of acquired data, is a powerful tool for exploring the chemical space of molecules. This approach is well-suited for fingerprinting purposes and solving different analytical tasks, including advanced second-order calibration and sample classification and/or discrimination [5,6,7].

Although the use of chromatographic fingerprints is usually fruitful, their effective processing and modeling is an on-going challenge. The retention process can be affected by unstable separation conditions. Thus, shifts of chromatographic peaks are an issue in the sample-to-sample comparisons. Differences in elution time of respective peaks are a source of undesired variability that hamper coming to proper conclusions. Therefore, before chemometric modeling of chromatographic data, the alignment of corresponding peaks is regarded as a necessary step. To date, many techniques for signal alignment were proposed [8]. Even though peak shifts can usually be effectively compensated across a collection of chromatographic fingerprints, it is a time-consuming procedure. Moreover, selecting a target signal, which is necessary to guide the alignment process, is not straightforward, especially if there are groups of samples in data [9]. The alignment of chromatographic fingerprints is a demanding task, regardless of the method considered. Thus, finding the possibility of avoiding this problematic preprocessing step is tempting and desired. Such a possibility arises when processing a second-order chromatographic fingerprint for any sample in the data, which was obtained from multichannel detectors as, for instance, a photodiode detector by the kernel Gram matrix [10,11]. The final data representation for a sample is a square and symmetric matrix but without the chromatographic dimension. To date, replacing the measurement data by its kernel representation has been mostly preferred in the context of speeding up calculations of different algorithms. While data variance is preserved, the kernel representation occupies less computer memory, and, eventually, the required computations are more efficient. This feature has been the primary motivation to think of kernel variants of multivariate chemometric techniques, resulting in developing the kernel variants of methods, such as principal component analysis [12] and partial least squares regression [13,14,15]. On the other hand, using a specific kernel, such as the radial basis or polynomial kernels, opens the unique possibility to perform nonlinear modeling, but using linear methods. This interesting advantage in the context of exploratory data analysis and modeling is known as the “kernel trick.” The “kernel trick” is also used in multiblock methods such as STATIS (Structuration des Tableaux À Trois Indices de la Statistique) [16] and multiple factor analysis (MFA) [17] in order to construct a common representation of blocks in which the same set of samples is described by a different type and number of variables. This common representation that is called “compromise” is calculated as a weighted sum of gram matrices for blocks.

In this article, we study in detail the kernels, which are less explored in the literature and which have the potential of modeling the second-order chromatographic data with peak shifts among samples. In several iterative scenarios, the efficiency estimates of the kernel-based approach concerning the ideal situation (no peak shifts in the chromatographic dimension) are obtained for differently simulated chromatographic three-way data. The performance of the kernel-based exploratory approach, called Gram PCA, is evaluated using figures of merit.

## 2. Theory

### 2.1. Representing the Second-Order Data for a Sample by a Kernel Gram Matrix

For a given *k*th sample (*k* = 1, …, *K*), the classic second-order chromatographic data, **X***_k_* (*I* × *J*), containing *I* (*i* = 1, …, *I*) chromatograms in rows (for a given wavelength) and *J* (*j* = 1, …, *J*) responses in columns (for a given elution time) of a multichannel detector (e.g., a spectrum), can be replaced by a representation, which is called the kernel Gram matrix. The kernel Gram matrix is defined as the product **X***_k_***X***_k_*^T^, where superscript T denotes the operation of the matrix transposition. The result is a symmetric and square matrix of dimensions (*I* × *I*). It is important to note that such an operation eliminates the chromatographic dimension (elution time) of the data. For a set of *K* samples, the final data can be organized as a three-way array **X** (*I* × *I* × *K*).

### 2.2. The Classic Variant of Principal Component Analysis

Principal component analysis (PCA) is a projection technique aiming to describe two-way data **X***_k_* (*I* × *J*) by a set of a few orthogonal latent variables, called principal components [18,19]. They are constructed to maximize the description of data variance. PCA is the bilinear decomposition model that can be represented as a product of *F* score vectors and *F* loading vectors, stored in columns of **T***_k_* (*I* × *F*) and **P***_k_* (*J* × *F*), respectively.

**X***_k_* = **T**_k_(**P**_k_)^T^ + **E***_k_* (1)

where **E***_k_* (*I* × *J*) is a matrix with residual elements (the elements that contain the information that was not explained by the PCA model with *F* principal components).

In fact, the projections of samples onto selected principal components are called scores, while loadings are the projections of variables onto selected PCs. A plot of one score vector against another is a score plot, which summarizes the multivariate data structure and displays the similarities among samples. A plot of two loading vectors (a loading plot) informs the contribution of every variable to the PCA model and reveals similar variables. In the majority of applications, the PCA technique supports the visualization of multivariate data. However, by maximizing the description of data variance, PCA is also considered a data compression technique with the help of which data information is summarized by a few principal components. Principal components replace the original explanatory variables and can serve as input to other data analysis methods. Such an approach speeds up the required calculations or even makes them possible such as during principal component regression.

### 2.3. Principal Component Analysis for Collection of Second-Order Data with Peak Shifts

As was mentioned earlier, compared to PCA, the Gram PCA approach uses the kernel Gram representation of the second-order chromatographic data (or a sample matrix) that was obtained from each sample. Thus, every sample data matrix is transformed to remove the chromatographic dimension into kernel Gram matrix to obtain **X** (*I* × *I* × *K*) prior to Gram PCA. Although the data can be arranged in a three-way array, the data analysis problem to be solved is described by the bilinear model defined by Equation (1). Similar to PCA, multivariate curve resolution (MCR) methods under various constraints can also be used for this purpose. The consecutive steps of the Gram PCA approach can be summarized as follows.

1. Unfold the *k*th (*k* = 1, …, *K*) sample Gram matrix (the *k*th slab of **X**) to obtain a vector of dimensions 1 × (*I × I*) and organize the vectors for all samples as *K* rows of the matrix **H** in order to obtain dimensions *K* × (*I* × *I*).
2. Construct the cross-product matrix, **S** = **HH**^T^.
3. Normalize matrix **S** to unit diagonal to obtain square and symmetric similarity matrix with the RV coefficients as elements [17,20].
4. Column center the similarity matrix.
5. Perform singular value decomposition (version of PCA) on the centered similarity matrix.

## 3. Results and Discussion

When different processes or systems are being studied, the overall conclusions are usually drawn based on the comparative analysis of samples or groups of samples formed in agreement with an experimental design. In particular, one is interested in revealing the differences among samples and explaining how the original data variables contribute to those differences. However, the major challenge arises with the need for comprehensive characterization of samples using instrumental techniques and extracting useful chemical information from the collected data. Chromatographic techniques can deliver comprehensive data, but the irreproducible retention processes can hamper their comparisons. It is generally known that retention of mixture components is very sensitive to the variability of experimental conditions in a given chromatographic system. Even minor fluctuations of column temperature, system pressure, ionic force, pH, the composition of the mobile phase, flow rate, and the on-going degradation of the stationary phase (the column aging) can induce massive shifts of peaks, and, in certain situations, may even change their elution order [21]. Sources of data variability affect the position of peaks and cannot be simply neglected when chromatographic signals, which describe samples are modeled as fingerprints. This modern approach is prevalent in the comparative analysis of complex samples and is preferred when the knowledge about sample composition is limited and/or chromatographic standards are not available. It is often used in metabolomic studies as an interesting alternative to targeted analysis. While the targeted analysis focuses on determining individual analytes, the fingerprinting approach (untargeted analysis) aims to reveal and explore as many components of a mixture as possible. In the latter variant of sample characterization, the position of chromatographic peaks and their correspondence across the collection of all signals is crucial, mostly when the differences among samples are effectively explained by chromatographic peaks of low intensity but strongly affected by the irreproducible retention process. As illustrated by simulations carried out in our study, this variability source was the most destructive, compared to the impact of other variability sources, such as instrumental noise (Gaussian and heteroscedastic noise) or baseline.

To illustrate to what extent the different data variability sources may affect first-order and second-order chromatographic signals and their interpretation, we considered the following data scenario. Simulated chromatographic profiles described two groups of samples with 30 samples each. They were formed due to two concentration levels of two out of thirteen mixture compounds. These two compounds were referred to as marker compounds. We assumed that the two groups summarize the basic discriminant problem, such as differentiating healthy and diseased patients or distinguishing between authentic and counterfeit products. Samples that were included into these two groups were simulated by allowing tiny random differences in the initial parameters (widths and heights) of chromatographic peaks. The initial settings of parameters were modified by 5% at most. Settings of parameters for two template chromatograms used to generate two groups of samples, including the positions of 13 chromatographic peaks, and their corresponding heights, widths, and areas, are listed in Table 1.

**Figure 1:**
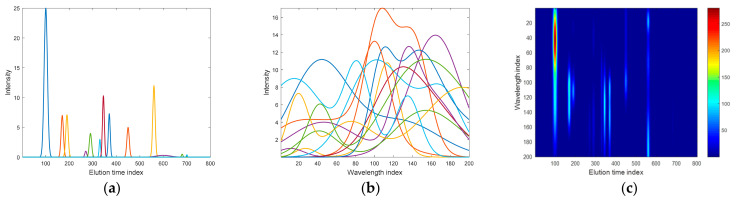
(**a**) Template of 13 signals of pure chromatographic peaks with 800 sampling points, (**b**) template of 13 pure spectral profiles containing 200 sampling points, and (**c**) a resulting second-order chromatographic signal for the exemplary sample from the first group.

All 13 chromatographic peaks strongly differed regarding their height, width, and elution behavior. The first marker component (peak no. 7) differentiated two groups of samples and eluted after ca. 345 sampling points. It had moderate intensity when compared to the remaining peaks. The difference in this peak intensity in the two groups of samples is relatively large and corresponds to ca. 30% (see Table 1). Therefore, this compound had a substantial contribution to the overall data variability. The second marker (peak no. 13) eluted very late, i.e., after ca. 700 sampling points. It was small and can hardly be spotted by the eye, even though there was a two-fold difference in its intensity in the two groups (see Table 1). It had excellent discriminative potential, but its impact on the total data variability was rather marginal. Pure chromatographic signals, pure spectral profiles, and the corresponding second-order chromatographic signal for the exemplary sample, which was selected from the first group, are presented in Figure 1.

**Figure 2:**
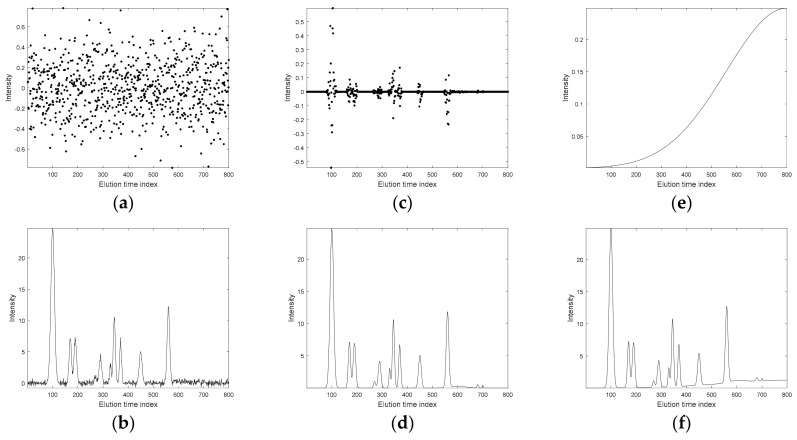
Effect of Gaussian noise, heteroscedastic noise, and baseline on the chromatogram of the first sample from a simulated data set: (**a**) simulated Gaussian noise and (**b**) chromatogram with Gaussian noise component, (**c**) simulated Gaussian noise (3.15% concerning the intensity of the largest peak), and (**d**) chromatogram with heteroscedastic noise component (2.4% concerning the intensity of the largest peak), (**e**) simulated baseline (5% concerning the intensity of the largest peak), and (**f**) chromatogram with a baseline.

Next, the noise and baseline components were added to the first-order pure chromatographic signals. For sample no. 1, the two types of noise (Gaussian and heteroscedastic) and baseline components that were considered are presented in Figure 2a,c,e. They introduced different effects. The baseline component changed the overall signal’s landscape and elevated chromatographic peaks with respect to the baseline intensity but had no effect upon peak shape.

**Figure 3:**
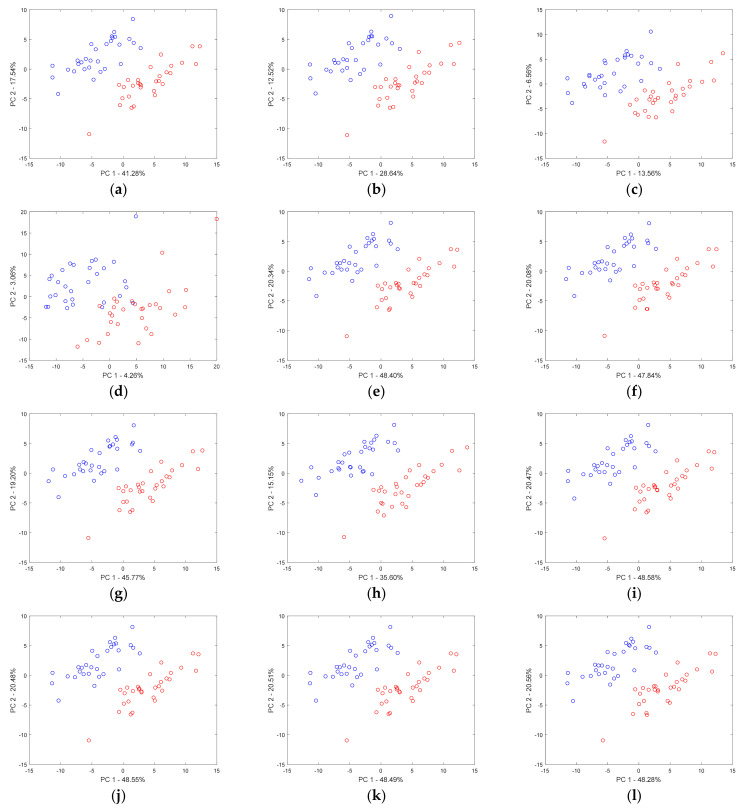
Projections of samples described by the first-order chromatograms on the space of the first two principal components: (**a**–**d**) the increasing level of Gaussian noise (4.34%, 8.67%, 17.35%, and 43.37% concerning the intensity of the largest peak, respectively), (**e**–**h**) the increasing level of heteroscedastic noise (2.39%, 4.78%, 9.56%, and 23.91% concerning the intensity of the largest peak, respectively), and (**i**–**l**) increasing level of baseline (0.5%, 1%, 2%, and 5% concerning the intensity of the largest peak, respectively).

In the consecutive sub-figures of Figure 3, the effect of Gaussian noise, heteroscedastic noise, and baseline upon the projection of samples in the space of the first two principal components is presented. A careful inspection of the respective sample projections allowed for concluding that, regardless of the type and level of influence for simulated chromatograms, none of these factors alone had a substantial effect. The major trend in the separation of the two groups of samples was preserved. The increase of the magnitude of Gaussian noise concerning the largest peak from ca. 4.3% up to ca. 43% influenced mainly the description of the overall data variability. In fact, the increase in the level of Gaussian noise enlarged the volume of data by stretching variables in each direction. The percentage of data variance explained by the first two principal components decreased. When the Gaussian noise was large, the two groups of samples started to overlap (see Figure 3d). Concerning the heteroscedastic noise, until its intensity corresponded to ca. 24% of the largest peak, both groups of samples were well separated in the space of two principal components. However, larger levels of heteroscedastic noise increased the overlap of groups. Different baseline levels within the tested range of intensity (i.e., up to 30% of the maximum of the highest peak) did not affect the clustering tendency unless the baseline component variability was not marginal.

**Figure 4:**
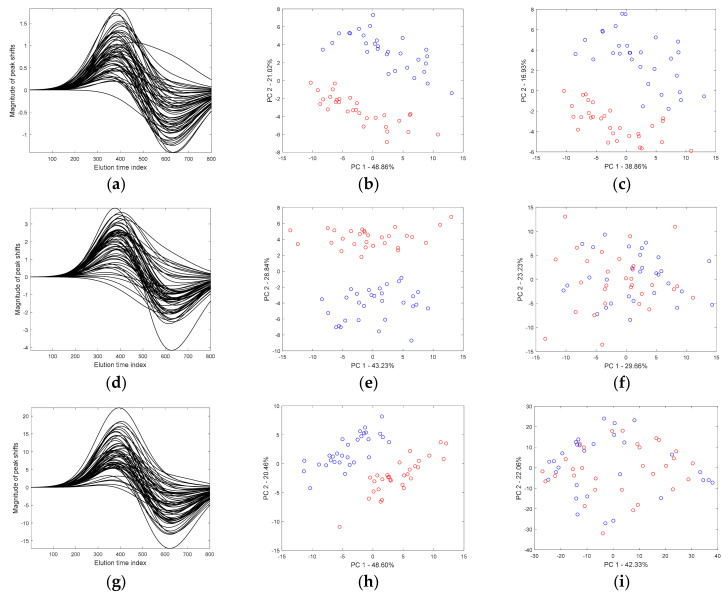
Simulated three sets of chromatographic signals containing two groups of samples. (**a**,**d**,**g**) warping functions. (**b**,**e**,**h**) projections of scores constructed using the Gram PCA approach on the space of the first two principal components for data without peak shifts. (**c**,**f**,**i**) the corresponding projections but for data with peak shifts.

Now, let us discuss the impact of peak shifts on the first-order (or a vector) chromatographic signals. In the presented scenario, we used a template of a strongly nonlinear warping function, which is depicted further on, in Section 4.5. This introduced peak shifts mostly in a region where the first marker was present (peak no. 7). Projections of samples on the space of the first two principal components, which is shown in the row sub-figures in Figure 4, correspond to the three simulated sets of chromatograms without peak shifts (middle column) and transformed using similar warping functions (the last column), respectively. The amplitude of warping functions was gradually increased from ca. 3, 8 up to 35 sampling points, as shown in Figure 4a,d,g. This, of course, caused larger peak shifts. The peak shifts that were expected to be very small, about three sampling points only, affected the clustering trend negligibly (compare two score projections shown in Figure 4b,c). This tendency can be maintained as long as the differences in signal intensities for consecutive sampling points in two groups of samples were explained effectively by the major principal components.

On the other hand, the separation between both groups of samples was poor. A stronger shifting of peaks, especially those located close to marker peak(s) and/or even coeluting with them, strongly amplified the variance, which was irrelevant. Then, with the increasing magnitude of anticipated peak shifts, the variability associated with the clustering tendency rapidly became weaker, and the ability to differentiate the two groups of samples by the first two principal components was lost rapidly. This happened already when peaks were expected to change their positions only by five additional sampling points compared to the first situation (analyze clustering trend in Figure 4c,f). As shown in Figure 4f, the additional variability associated with the peak shift pattern, which was defined by the warping function, finally overwhelmed the major separation trend. That was why the first two principal components no longer explained the clustering tendency. From the PCA perspective and the construction of the principal components, this means that the overall data variability has substantially changed. Thus, the principal components were rotated adequately to capture this trend to the largest possible extent.

**Figure 5:**
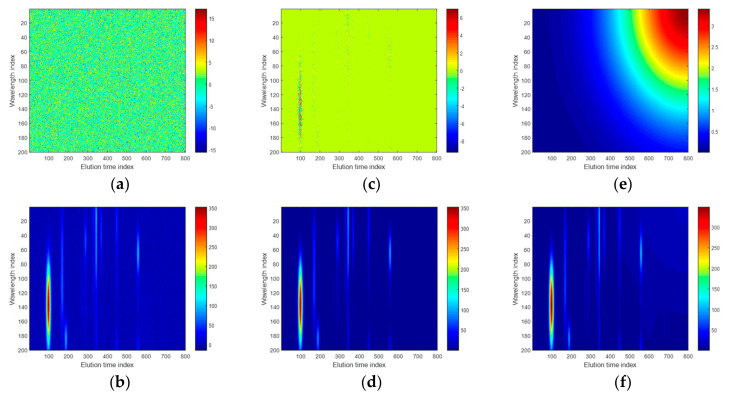
(**a**) Simulated Gaussian noise and (**b**) resulting second-order chromatographic signal, (**c**) simulated heteroscedastic noise, and (**d**) resulting second-order chromatographic signal, (**e**) simulated baseline, and (**f**) resulting second-order chromatographic signal.

The next simulation study deals with some additional data variability sources and their impact on the discriminant power of two groups of samples, which were characterized by the second-order chromatographic signals. In Figure 5, an exemplary simulated noise and baseline components with the resulting second-order (or a matrix) chromatographic signal for a sample are presented as color maps. The color maps display the elements of a data matrix as colored pixels. Their color intensity and hue are selected from a given color scale (see the color bar in Figure 5). Elements that are described by low values are indicated as navy (cold) pixels, whereas those having large values correspond to red (hot) pixels. As illustrated in Figure 5b,d,f, the magnitude of noise level and baseline signal were not larger than ca. 4% concerning the largest intensity, which was observed in the pure chromatographic signal, but all of the variability sources contributed to the overall landscape of the second-order signal.

**Figure 6:**
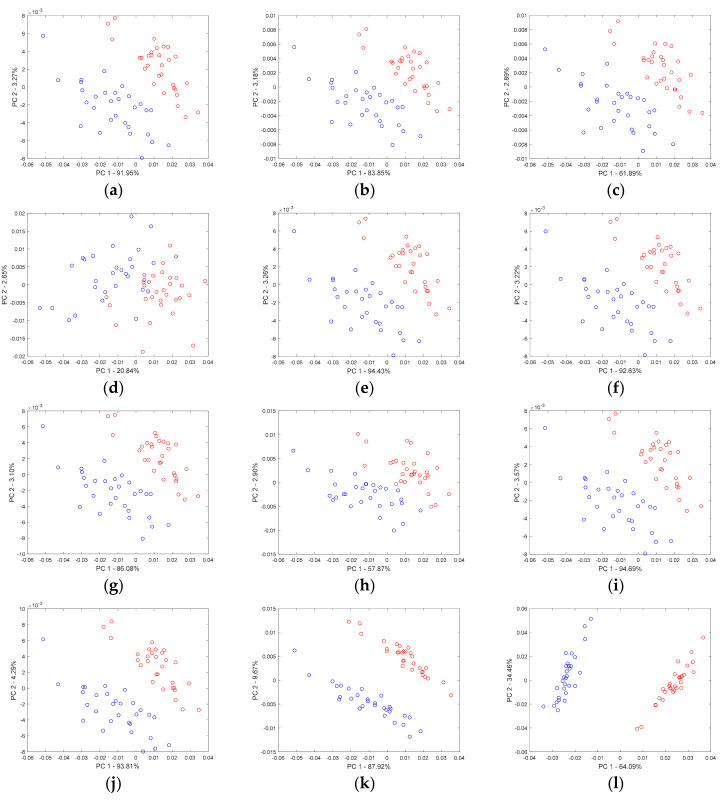
Projections of samples described by one-dimensional chromatograms in the space of the first two principal components: (**a**–**d**) the increasing level of Gaussian noise (2.27%, 4.54%, 9.08%, and 22.7% concerning the intensity of the largest peak, respectively), (**e**–**h**) the increasing level of heteroscedastic noise (1.68%, 3.36%, 6.72%, and 16.8% concerning the intensity of the largest peak, respectively), and (**i**–**l**) increasing level of baseline (0.54%, 1.07%, 2.14%, and 5.35% concerning the intensity of the largest peak, respectively).

For the exemplary data set, we investigated the potential impact of noise and baseline on the distribution of samples in the space of the first two principal components obtained from samples represented by the Gram matrices. With the increasing magnitude of Gaussian noise by a factor of 0.5, 1, or 2, the clustering trend observed on the first two principal components’ score plots that remained robust against peak shifts. However, the increasing Gaussian noise affected the proportion between two variability sources, namely the portion of variance explaining the clustering trend and an additional variance, which was more associated with the noise. This reasoning finds confirmation in the decreasing percentage of modeled data variance. For the original data, the first principal component accounted for 95.04% of the total data variability. When the noise level increased by a factor of 0.5, 1, or 2, the amount of information, which was described by the first principal component corresponding to 91.95%, 83.85%, and 61.89%, respectively. Once the Gaussian noise level became very large (i.e., it was amplified by a factor of 5), the separation of groups of samples on the first two principal components was not defined well. For this situation, the total data variance explained by the first principal component accounted only for 20.84%. A similar tendency was observed for the pure second-order chromatographic signals when they were contaminated with different levels of heteroscedastic noise (see Figure 6e–h). The amount of explained data variability dropped rapidly with the increase of the heteroscedastic noise level, but, surprisingly, even for a relatively large level of heteroscedastic noise, the general separation trend between the two groups of samples was preserved. The distribution of samples in the first two principal components’ space was virtually the same as the distribution of samples described by the set of pure second-order chromatographic signals. For the largest level of heteroscedastic noise, which was considered in this study, the total sum of the squared Euclidean distances computed in the space of the first two principal components for the respective samples represented by the pure and contaminated (with heteroscedastic noise) signals equals 5.3 × 10^−4^.

The last case illustrates the effect of increasing baseline intensity. Consecutive sub-plots of Figure 6j,k,l revealed a rather unexpected contradiction for the tendency, which was observed for the increasing noise levels. Namely, for this data set, a more intense baseline enhanced the separation between the two groups of samples. This striking conclusion can be explained by the shape of the simulated baseline template and its beneficial influence on the peak’s intensity corresponding to the second marker compound eluting after ca. 700 sampling points (see Figure 1a). Compared to the role of the first marker compound, the contribution of this peak to the overall data variance and modeling differences between two groups of samples was marginal so far. However, elevating the second discriminant peak intensity enhanced the variability and, thus, supported the discriminant power, and the separation between the two groups of samples and their projections on the first two principal components became distinctive. Moreover, the scatter of samples in each group decreased, i.e., the within-group variance shrank strongly.

**Figure 7:**
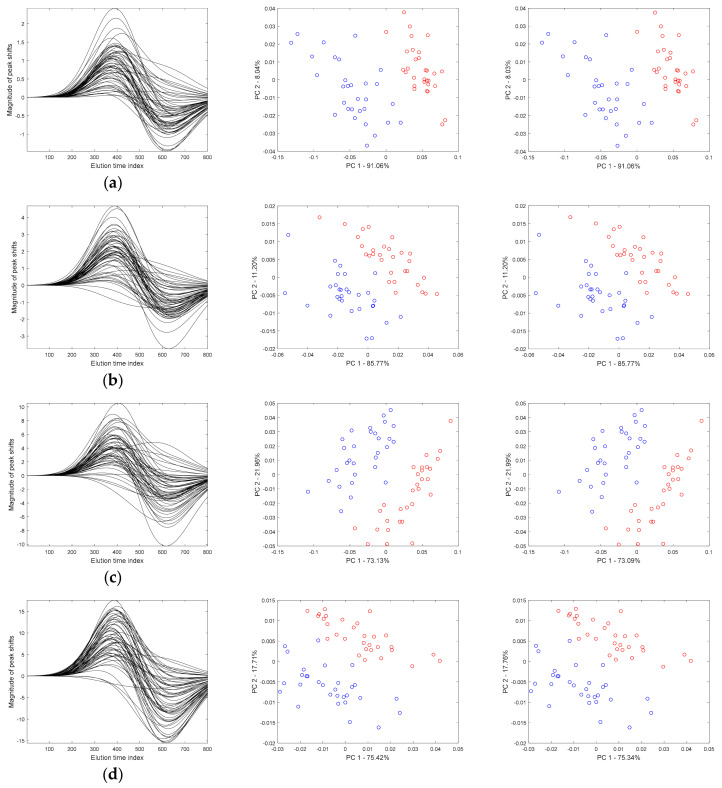
Set of warping functions and the corresponding projections of samples described by second-order chromatograms without peak shifts and with peak shifts on the plane defined by the first two principal components obtained from the Gram PCA approach, respectively, for four different levels of allowed peak shifts up to (**a**) three sampling points, (**b**) nine sampling points, (**c**) 20 sampling points, and (**d**) 33 sampling points.

Now, let us discuss the effect of peak shifts and their influence on the overall interpretation of the score plots constructed using the Gram PCA approach. For this purpose, four different sets of “pure“ second-order chromatographic signals were simulated. Then, the chromatographic peaks were transformed to induce peak shifts. Positions of peaks were adjusted according to the warping functions simulated using the second template of the warping function in order to obtain extreme and nonlinear peak shifts (see Figure 7), especially in the region where the first marker peak was present. In the consecutive row sub-plots of Figure 7, the corresponding set of warping functions, distribution of samples in the space of the first two principal components for original second-order signals (the “ground truth” data), and signals with peak shifts are presented. The two score projections were constructed using the Gram PCA approach.

Even though the magnitude of allowed peak shifts in signals increased (from ca. 3 up to 33 sampling points), it did not affect the distribution of samples observed on the first two principal components. The Euclidean distances between corresponding samples in the two projections constructed for original and transformed signals were very small, which is almost negligible. It is important to emphasize that, in the extreme case presented in Figure 7d, peaks were allowed to change their positions concerning the original signal’s position up to ca. 33 sampling points. Principal components constructed using the Gram PCA approach were not affected by peak shifts, even the large ones, and the general trend is preserved in the score plots (compare middle and left-hand side image in Figure 7d). In fact, this unique property is solely due to the second-order advantage. At this point, it is relevant to acknowledge the support of information explained by the spectral profiles. When spectral profiles differentiate various mixture components well, the peak shifts, which were observed along the chromatographic data dimension, can be effectively handled. As illustrated by score plots shown in Figure 7, a comparison of second-order chromatographic signals was possible without their preliminary alignment. The collection of second-order signals was transformed into Gram matrices and then analyzed using the Gram PCA approach to reveal similarities among samples in the principal components’ space.

In this part of the discussion section, we are going to present the results of a large-scale simulation study obtained for 100 generated data sets. They were constructed in the iterative process to facilitate evaluating the potential effect caused by the peak shifts upon the interpretation of the score plots that were constructed using the Gram PCA approach.

Two different nonlinear templates of warping functions were used to transform chromatographic peaks of simulated second-order signals. Results were collected in the iterative process in which the impact of warping functions was gradually increased by multiplying elements of each function by a factor in the range of 0.1 to 1, with a step of 0.1. 

**Figure 8:**
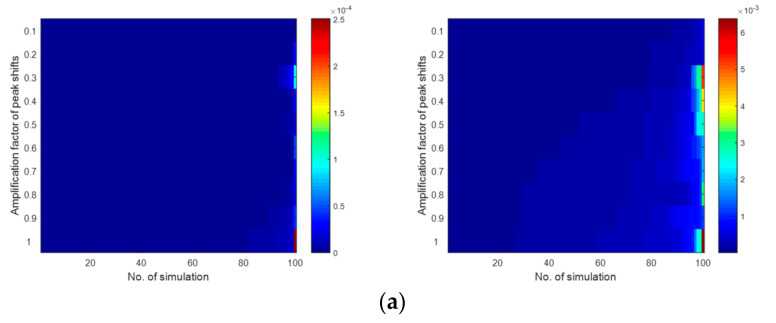

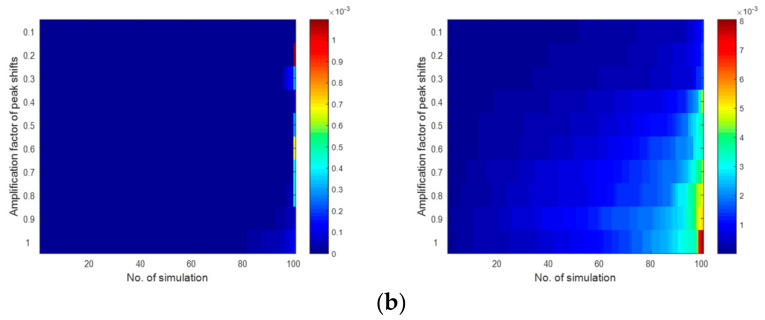
Color maps presenting sorted results of simulation study taking into account gradually increasing peak shifts in signals for two different templates of warping functions, respectively: (**a**) absolute differences computed between coordinates of the corresponding samples described by second-order signals and their variant with peak shifts induced by two different templates of warping functions, (**b**) absolute differences computed between the percentage of explained data variance by corresponding principal components obtained from the Gram PCA approach for original and transformed data using two different templates of warping functions.

In Figure 8, the results are presented in the form of colormaps. They were constructed for two different templates of warping functions. Colormaps summarized the results obtained from each iteration for a given amplification factor of peak shifts and were arranged in each consecutive row from the smallest to the largest. Each colormap element corresponded to the Euclidean distance, which was computed between the corresponding samples from the original and transformed data or the sum of the absolute differences in the percentage of data variance explained between the respective principal components (the second column in Figure 8). The analysis of the colormaps allowed us to conclude that, for simulated data sets, the solution of the Gram PCA is stable. Potential differences between score plots constructed for the original set of second-order chromatograms and transformed ones are relatively negligible. Nonetheless, two characteristic patterns were revealed in this simulation study and are worth mentioning. Namely, as was expected, the shape of the warping function had an impact. For the simpler nonlinear template of warping function, the average differences, which were observed among samples in the space of principal components, were ca. by one order of magnitude smaller than the results obtained when chromatograms were transformed using the second template with strongly nonlinear warping functions. Moreover, with the increasing impact of the warping functions, in general, their influence upon scores was also substantially larger.

In the last large-scale iterative study, we examined the impact of severe peak shifts when constructing principal components for the kernel Gram data representation and their interpretation. 

**Figure 9:**
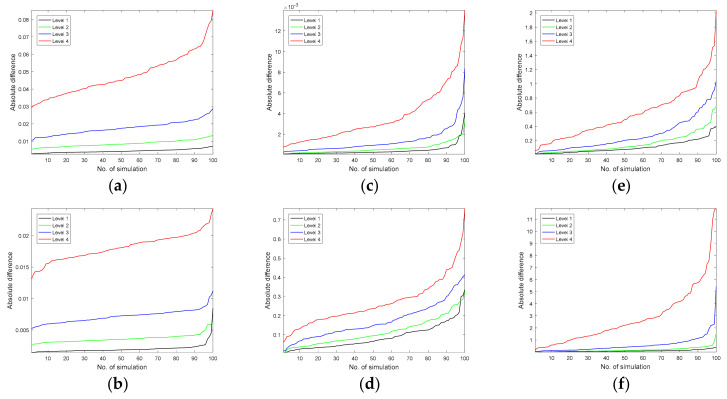
Sorted absolute average differences, obtained for 100 different simulations, calculated between the corresponding samples in the space of principal components constructed using the Gram PCA approach for original second-order chromatographic signals and data with peak shifts and: (**a**) four levels of Gaussian noise concerning the largest observed peak (level 1: 2.27%, level 2: 4.54%, level 3: 9.08%, and level 4: 22.7%), (**b**) four levels of heteroscedastic noise concerning the largest observed peak (level 1: 1.68%, level 2: 3.36%, level 3: 6.72%, and level 4: 18.8%), (**c**) four levels of baseline concerning the largest observed peak (level 1: 0.54%, level 2: 1.07%, level 3: 2.14%, and level 4: 5.35%). Subplots (**d**,**e**), and (**f**) present sorted average differences in explained data variance by the corresponding principal components constructed using the Gram PCA approach for original chromatographic signals and signals with peak shifts and obtained for the same situations and levels as in subplots (**a**–**c**).

By using the second template of the warping function, amplified by a factor of five, peaks are allowed to change their positions to a large extent, i.e., up to ca. 35 sampling points concerning the corresponding peaks in the original chromatographic signals. During the iterative study, either Gaussian noise, heteroscedastic noise, or baseline was added to the simulated and transformed second-order chromatographic signals at four different amplification levels. Sub-plots in the first row of Figure 9 present the sorted sums of absolute differences calculated between coordinates of corresponding samples in the space of principal components for perturbed chromatographic signals either with Gaussian noise, heteroscedastic noise, or baseline and signals with peak shifts. In the second row of Figure 9, the sorted sums of absolute differences between explained data variance by the consecutive principal components are displayed for the same three scenarios. In general, with the increasing levels of signal contamination, differences among corresponding samples were becoming larger. However, this had no real impact on the interpretation of obtained score projections, even for a relatively large contamination level. In the simulation study, the maximum level of contamination for the Gaussian noise, heteroscedastic noise, and baseline reached ca. 23%, 17%, and 5.5%, respectively, with respect to the intensity of the largest observed peak in the data. Score plots displaying distributions of samples described by the second-order chromatographic signals, which were affected by peak shifts and supplemented with different noise levels or baselines were virtually the same with the score plots obtained for the “ground truth” signals (i.e., not affected by peak shifts, but noisy). A similar tendency was observed in Figure 9b,d,f. With the increasing level of contamination, the absolute differences calculated between the amount of variance explained by principal components for the “ground truth” data and the corresponding data but affected by peak shifts became larger.

## 4. Materials and Methods

### 4.1. A Simulation Study

During the iterative simulation of data, multiple pairs of second-order chromatographic data sets were generated using a given template of chromatographic peaks and respective spectral profiles. The first data set served as the ‘ground truth’ or as a reference data set, which was used to evaluate the efficiency of the proposed approach. This collection of chromatographic signals was without peak shifts. The second data set contained the same chromatographic Gaussian peak profiles. The widths and heights and the spectral profiles of chromatographic data were the same as in the reference data, but the corresponding peaks were perturbed using the warping function. Different sources of experimental variability were simulated at each iteration, i.e., various changes in peak heights, widths, and fluctuations of signals baseline, to mimic real data. Later on, different levels and types of noise components and different baseline landscapes with a different overall intensity were included and further discussed.

### 4.2. Simulated Chromatographic Data

For the sake of simplicity, the concentration profiles of *F* components were represented by *F* Gaussian peaks. Their heights, widths, and the position along the elution time axis containing *J* sampling points were a priori defined. The *F* spectral profiles, describing each component, were simulated as a mixture of three Gaussian peaks. Their heights, widths, and locations along the wavelength axis, containing *I* sampling points, were allowed to change randomly with a specified degree of uncertainty. The final second-order chromatographic data, describing a given sample data matrix, **X***_k_*, had *I* rows (chromatograms measured at the *i*th wavelength channel) and *J* columns (spectra of portions of mixture eluting from a column at a given elution time). It was obtained as a product of a matrix **T***_k_* (*I* × *F*) with *F* pure concentration profiles in columns, and matrix **P***_k_* (*J* × *F*) with *F* pure spectral profiles in columns. The model is the same as described by Equation (1), but in contrast to PCA, there was not an orthogonality constraint for the columns of **T***_k_* and **P***_k_*, and the pure concentration and spectral profiles were constrained to be non-negative.

### 4.3. Simulating the Baseline Component of the Second-Order Chromatographic Data

The baseline component was generated using the bilinear model given by Equation (2). The resulting data matrix with the baseline component, **B**, has the same dimensions as the pure chromatographic signal. It contained a single Gaussian peak in the chromatographic and spectral domains. The baseline component elevated the pure chromatographic signals. However, depending on the selected landscape of the baseline, the elevated effect was expressed differently along the chromatographic dimension. In the spectral domain, the influence of baseline was also different and mimicked the very low absorption of the mobile phase.

### 4.4. Simulating the Noise of the Second-Order Chromatographic Signal

In the simulations, two noise scenarios were considered, namely, Gaussian noise (fixed amplitude of noise) and the heteroscedastic noise (amplitude of the noise was proportional to the signal’s intensity). The Gaussian noise and heteroscedastic noise were drawn from the normal distribution with zero mean and unit standard deviation, *N*(0,1).

### 4.5. Simulating Two Different Templates for the Warping Functions

**Figure 10:**
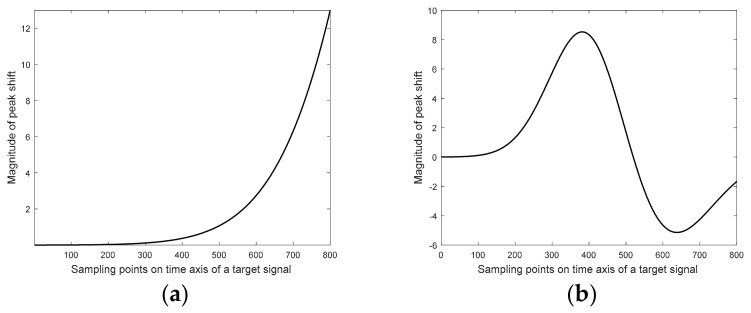
Two templates of warping functions: (**a**) smooth and nonlinear and (**b**) smooth and strongly nonlinear.

The shape of the warping function was modeled using a mixture of two Gaussian peaks. Their parameters, such as position, height, and width, were modified slightly at each simulation study step. Two templates of warping functions were considered. They varied in terms of nonlinearity, and, thus, the correction of peak shifts in signals requires a substantial effort. The first template of the warping function, depicted in Figure 10a, was smooth and nonlinear.

The magnitude of peak shifts was increasing with the elution time in a nonlinear manner. Thus, the largest peak shifts were observed when compounds (simulated peaks) eluted late. The second template of the warping function mimicked a situation when peak shifts were strongly perturbed, and the elution behavior was very different compared to the first scenario. At the beginning of the separation process, peak shifts were negligible. Peaks eluting during the first 500 sampling points appeared later than the respective peaks, which were observed in the target chromatogram (see Figure 10b). According to the model, which described the peak shifts, a delay of elution for chromatograms appearing after 380 sampling points was expected to be the most considerable. Later on, the delay in the elution process was becoming smaller. Eventually, the separation of peaks was getting faster up to ca. 650 sampling points, and, later, started to slow down.

## 5. Conclusions

Elimination of undesired data variation sources before any comparative analysis of samples, in particular variability of chromatographic conditions, enhances the true analytical signal and, thus, improves the overall data quality. Eventually, the comparative analysis of samples can be focused on the underlying differences among groups of samples. Principal components and the corresponding projections of samples constructed using the Gram PCA, when they are described by second-order chromatographic signals, are robust in terms of resistance to potential peak shifts.

## Figures and Tables

**Table 1:** Detailed information about chromatographic peaks (location, height, width, and area) contributing to template chromatographic signals used to generate two groups of samples.

Group	Peak No.	1	2	3	4	5	6	7	8	9	10	11	12	13
**1**	**center**	100	170	190	270	290	330	345	370	450	560	600	680	700
	**height**	25	7	7.1	1	4	3	10.3	7.3	5	12	0.3	0.5	0.4
	**width**	7	5	5.1	4	5	3	4	4	5	5	30	3	2
	**area**	438.66	87.73	90.77	10.03	50.13	22.56	103.27	73.19	62.67	150.40	22.56	3.76	2.01
**2**	**center**	100	170	190	270	290	330	345	370	450	560	600	680	700
	**height**	25	7	7.1	1	4	3	7.3	7.3	5	12	0.3	0.5	0.2
	**width**	7	5	5.1	4	5	3	4	4	5	5	30	3	2
	**area**	438.66	87.73	90.77	10.03	50.13	22.56	73.19	73.19	62.67	150.40	22.56	3.76	1.00

## Data Availability

Data sets used in the study were simulated as described in the section “Materials and Methods”.

## Sample Availability

Not available.
